# Supplementary material for: Is the Hitchcock Story Really True? Public Opinion on Hooded Crows in Cities as Input to Management
Source: Animals (Basel). 2022 May 7;12(9):1207. doi: 10.3390/ani12091207 (PMC9105359; doi:10.3390/ani12091207)
Supplement: Supplementary file 1 [file animals-12-01207-s001.zip › Supplementary File 1.pdf]

Table S2. Demographic and resident data of the respondents (N = 1740).

Demographic Variable	Levels	N	%
Gender	Women	1276	73.3
	Men	464	26.7
Age	Below 19 years	75	4.3
	Between 20 and 39 years	1055	60.6
	Between 40 and 59 years	509	29.3
	Above 60 years data	101	5.8
Highest level of education	Primary school	38	2.2
	Secondary school	727	41.8
	High vocational edication or BS degree	684	39.3
	Master's degree/PhD degree	291	16.7
Place of residence	Capital	212	12.2
	County town	852	49.0
	Urban	375	21.5
	Rural	301	17.3
Workplace	Capital	290	16.6
	County town	920	52.9
	Town	384	22.1
	Rural	146	8.4
Membership in hunting association	Yes	27	1.6
	No	1713	98.4
Membership in nature conservation or animal welfare organization	Yes	169	9.7
	No	1571	90.3
