# Supplementary material for: Is the Hitchcock Story Really True? Public Opinion on Hooded Crows in Cities as Input to Management
Source: Animals (Basel). 2022 May 7;12(9):1207. doi: 10.3390/ani12091207 (PMC9105359; doi:10.3390/ani12091207)
Supplement: Supplementary file 1 [file animals-12-01207-s001.zip › Supplementary File 2.pdf]

For two species, correct identification depended on the gender of the respondents. The Hooded Crow was correctly identified by 82% of men and 74% of women ($\chi^2 = 11.088$, df = 1, $p < 0.001$, $\eta\hat{}^2$: 0.0064). In contrast, the Rook was correctly identified by 66% of men and 58% of women ($\chi^2 = 8.691$, df = 1, $p = 0.003$, $\eta\hat{}^2$: 0.005). Age had no effect on species knowledge except in cases of Magpie ($\chi^2 = 16.823$, df = 3, $p = 0.001$, $\eta\hat{}^2$: 0.0097) and Jay ($\chi^2 = 12.55$, df = 3, $p = 0.006$, $\eta\hat{}^2$: 0.0072) because older age groups were more successful in recognizing these species.
