# Supplementary material for: Is the Hitchcock Story Really True? Public Opinion on Hooded Crows in Cities as Input to Management
Source: Animals (Basel). 2022 May 7;12(9):1207. doi: 10.3390/ani12091207 (PMC9105359; doi:10.3390/ani12091207)
Supplement: Supplementary file 1 [file animals-12-01207-s001.zip › Supplementary materials_Captions_LKetal..pdf]

Is the Hitchcock Story Really True? Public Opinion on Hooded Crows in Cities as Input to Management

László Kövér [1,*], Petra Paládi [1,2], Isma Benmazouz [1,2], Andrej Šorgo [3,4], Natalija Špur [3], Lajos Juhász [1], Péter Czine [5], Péter Balogh [5] and Szabolcs Lengyel [6]

1 Department of Nature Conservation, Zoology and Game Management, University of Debrecen, Boszormenyi str. 138., Debrecen 4032, Hungary; paladi.petra@agr.unideb.hu (P.P.); benmazouz.isma@agr.unideb.hu (I.B.); juhaszl@agr.unideb.hu (L.J.)
2 Doctoral School of Animal Science, University of Debrecen, Boszormenyi str. 138., Debrecen 4032, Hungary
3 Faculty of Natural Sciences and Mathematics, University of Maribor, Koroska cesta 160, Maribor 2000, Slovenia; andrej.sorgo@um.si (A.Š.); natalija.spur@um.si (N.Š.)
4 Faculty of Electrical Engineering and Computer Science, University of Maribor, Koroska cesta 46, Maribor 2000, Slovenia
5 Department of Economic Analysis and Statistics, University of Debrecen, Boszormenyi str. 138., Debrecen 4032, Hungary; czine.peter@econ.unideb.hu (P.C.); balogh.peter@econ.unideb.hu (P.B.)
6 Department of Tisza Research, Institute of Aquatic Ecology, Centre for Ecological Research, Eötvös Loránd Research Network, Bem ter 18/c, Debrecen 4026, Hungary; lengyel.szabolcs@ecolres.hu
* Correspondence: koverl@agr.unideb.hu

Supplementary materials

Supplementary material, Figure S1: Mean score of agreement with statements on coexisistence with Hooded Crows by the gender of the respondents. Only 14 statements are shown for which the gender difference was significant (p<0.01).

Note: For management of crow numbers, all measures by experts are acceptable. 4: I find measures to control crow populations acceptable and support them. 5: Damages caused by crows are minor and do not justify population control measures. 6: The number of crows should be reduced regardless of the type of their habitat. 7: Crows should not be bothered as their numbers will reach a natural balance. 8: We should protect crows regardless of the type of their habitat. 9: I would like to participate in projects aiming to control the population of crows. 10: The Hooded Crow is just one of many bird species that should enjoy unlimited protection. 12: Damages caused by crows should be reimbursed but should not justify population control. 13: Crows should be de-listed as game species, which would make their protection easier. 15: City crows should enjoy unlimited legal protection. 16: Claims to control populations come from the hunting lobby, who aim to shoot more crows. 19: Crow population control is beyond my scope and should be the business of experts. 21: I am indifferent to crows, I have no interest in them or any problem with them.

Supplementary material, S2: Crow survey questionnaire

Supplementary material, S2: Certificate of he ethics committee
