# Supplementary figures and images for: Is the Hitchcock Story Really True? Public Opinion on Hooded Crows in Cities as Input to Management
Source: Animals (Basel). 2022 May 7;12(9):1207. doi: 10.3390/ani12091207 (PMC9105359; doi:10.3390/ani12091207)

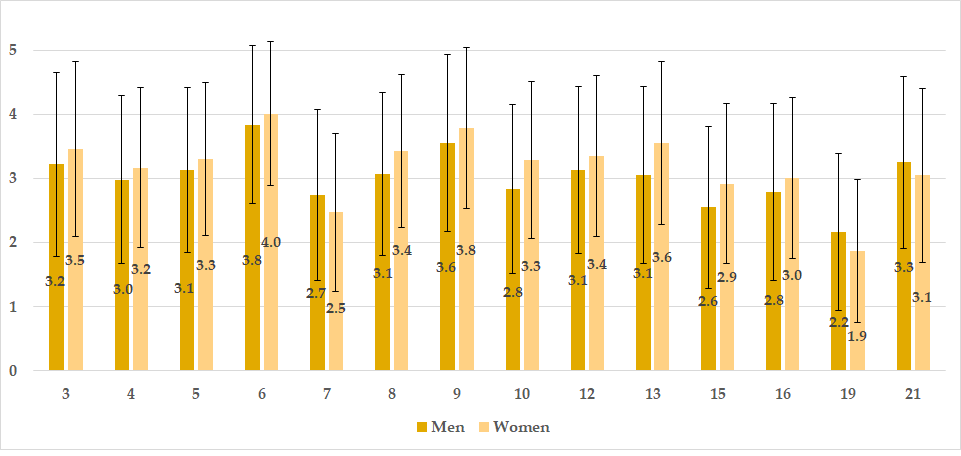

Supplement: Supplementary file 1 [file animals-12-01207-s001.zip › The following supporting information can be downloaded at_ www.mdpi.com/xxx/s1, Figure S1_ title]
