# Supplementary material for: Is the Hitchcock Story Really True? Public Opinion on Hooded Crows in Cities as Input to Management
Source: Animals (Basel). 2022 May 7;12(9):1207. doi: 10.3390/ani12091207 (PMC9105359; doi:10.3390/ani12091207)
Supplement: Supplementary file 1 [file certificate_Etics_Committee.pdf › certificate_Etics_Committee.pdf]

UNIVERSITY of DEBRECEN

FACULTY OF AGRICULTURAL AND FOOD SCIENCES AND
ENVIRONMENTAL MANAGEMENT
DEAN
H-4032 Böszörményi 138, Debrecen
Tel/fax: +3652/508-412, email: mekdekania@agr.unideb.hu

Dear Dr. Kövér,

Your project/manuscript entitled "*Is the Hitchcock story really true? Public opinion on Hooded crows as a necessary tool of management*" was reviewed by the Faculty's Ethics Committee and a waiver of informed consent is granted. A full waiver of HIPAA authorization is also granted for your research, as we concluded that the IEB can issue the waivers because this research involves no more than minimal risk to the privacy of participants and falls into the following exempt category: "**Research involving the use of educational tests (cognitive, diagnostic, aptitude, achievement), survey procedures, interview procedures or observation of public behavior**".

Sincerely,

Dr. László Stündl Ph.D.

Dean of Faculty of Agricultural

and Food Sciences and Environmental Management

Debrecen, 31.03.2022
